# Zanidatamab vs trastuzumab with or without PD-1 blockade in HER2-positive metastatic gastric cancer: a reconstructed patient-level pooled analysis

**DOI:** 10.1093/oncolo/oyag270

**Published:** 2026-07-14

**Authors:** Federico Nichetti, Floriana Nappo, Francesca Bof, Martina Bosa, Elena Mattiuzzo, Giulia Maddalena, Maria Caterina De Grandis, Sabina Murgioni, Francesca Bergamo, Sara Lonardi

**Affiliations:** Medical Oncology 1, Veneto Institute of Oncology IOV-IRCCS, Padua, 35128, Italy; Department of Surgery, Oncology and Gastroenterology, University of Padua, Padua, 35122, Italy; Medical Oncology 1, Veneto Institute of Oncology IOV-IRCCS, Padua, 35128, Italy; Medical Oncology 1, Veneto Institute of Oncology IOV-IRCCS, Padua, 35128, Italy; Department of Surgery, Oncology and Gastroenterology, University of Padua, Padua, 35122, Italy; Medical Oncology 1, Veneto Institute of Oncology IOV-IRCCS, Padua, 35128, Italy; Department of Surgery, Oncology and Gastroenterology, University of Padua, Padua, 35122, Italy; Medical Oncology 1, Veneto Institute of Oncology IOV-IRCCS, Padua, 35128, Italy; Medical Oncology 1, Veneto Institute of Oncology IOV-IRCCS, Padua, 35128, Italy; Medical Oncology 1, Veneto Institute of Oncology IOV-IRCCS, Padua, 35128, Italy; Medical Oncology 1, Veneto Institute of Oncology IOV-IRCCS, Padua, 35128, Italy; Medical Oncology 1, Veneto Institute of Oncology IOV-IRCCS, Padua, 35128, Italy; Medical Oncology 1, Veneto Institute of Oncology IOV-IRCCS, Padua, 35128, Italy

**Keywords:** gastric cancer, HER2, trastuzumab, zanidatamab, pembrolizumab, pooled analysis

## Abstract

**Background:**

First-line treatment of HER2-positive advanced gastric/GEJ adenocarcinoma now includes pembrolizumab added to trastuzumab plus chemotherapy (KEYNOTE-811). Zanidatamab, a bispecific anti-HER2 antibody, improved outcomes in HERIZON-GEA-01 but has not been directly compared with this standard.

**Methods:**

We searched PubMed, Scopus, and the ASCO/ESMO libraries for phase II/III trials of previously untreated HER2-positive metastatic gastric/GEJ adenocarcinoma. Individual patient data were reconstructed from published Kaplan-Meier curves. Cox frailty models estimated hazard ratios using the pembrolizumab regimen as the reference. Restricted mean survival time analysis was performed at 12, 24, and 36 months.

**Results:**

Nine trials were included: trastuzumab + chemotherapy (*n* = 1466), pembrolizumab + trastuzumab + chemotherapy (*n* = 430), zanidatamab + chemotherapy (*n* = 350), and zanidatamab + tislelizumab + chemotherapy (*n* = 335). Median PFS was 12.5 months with both zanidatamab regimens vs 10.0 months with pembrolizumab. The zanidatamab-tislelizumab triplet significantly reduced progression hazard (HR, 0.78, 95% CI, 0.62-0.99; *P* = 0.042). Median OS was 27.0 and 25.3 months with zanidatamab arms vs 20.3 months with pembrolizumab. Both zanidatamab arms showed significantly longer OS at 36 months by RMST.

**Conclusions:**

Zanidatamab-based regimens were associated with longer PFS and OS than the pembrolizumab standard, though direct randomized comparisons are needed.

Implication for Practice While direct comparisons between zanidatamab and pembrolizumab regimens in HER2-positive gastric cancer are lacking, this pooled analysis suggests that bispecific HER2 blockade is a highly competitive alternative. The zanidatamab-tislelizumab triplet significantly reduced the risk of progression versus pembrolizumab (HR, 0.78; *P* = .042), and both zanidatamab arms achieved significantly longer overall survival at 36 months by restricted mean survival time analysis. Practically, these findings highlight the potential of intensified HER2 targeting to improve long-term outcomes, though direct randomized trials are needed to definitively confirm these indirect results.

## Introduction

The therapeutic landscape for HER2-positive advanced gastric and gastroesophageal junction (GEJ) adenocarcinoma has evolved significantly in recent years.^1^ For over a decade, the treatment paradigm was defined by the landmark ToGA trial, which established trastuzumab in combination with fluoropyrimidine- and platinum-based chemotherapy as the first-line standard.^2^ This phase III study demonstrated a significant survival benefit, extending median overall survival (OS) from 11.1 to 13.8 months. Since then, extensive academic efforts have focused on refining patient selection and identifying mechanisms of primary or secondary resistance to trastuzumab; however, these findings have yet to alter clinical practice. Similarly, multiple trials investigating novel anti-HER2 combinations or sequences failed to meet their primary endpoints, leaving clinical outcomes largely modest.

The next major breakthrough arrived with the integration of immune checkpoint inhibitors (ICIs) into HER2-targeted regimens.^3^ The phase III KEYNOTE-811 trial evaluated the addition of pembrolizumab to trastuzumab and chemotherapy in the first-line setting. Initial analyses showed significant improvements in objective response rate (ORR) and progression-free survival (PFS).^4^ The final OS analysis confirmed a significant benefit, with a median OS of 20.0 months in the pembrolizumab arm versus 16.8 months in the control arm.^5^ Notably, this benefit was restricted to tumors with a PD-L1 Combined Positive Score (CPS) ≥1 (approximately 85% of cases), whereas those with CPS < 1 derived no additional benefit. Consequently, contemporary guidelines now recommend the triplet of chemotherapy, trastuzumab, and pembrolizumab as the preferred first-line therapy for HER2-positive advanced disease with a PD-L1 combined positive score (CPS) ≥1.^6^

Despite these advances, resistance to HER2-targeted therapy remains a challenge, driving the development of next-generation agents. Zanidatamab is a novel bispecific antibody that targets 2 distinct extracellular domains of the HER2 receptor. This biparatopic binding induces receptor clustering, enhances internalization, and potentiates immune-mediated tumor cell killing. The phase III HERIZON-GEA-01 trial compared zanidatamab plus chemotherapy (with or without the PD-1 inhibitor tislelizumab) against the ToGA regimen (trastuzumab plus chemotherapy). At the primary study analysis, both zanidatamab-containing arms significantly improved PFS. Furthermore, the combination of zanidatamab, tislelizumab, and chemotherapy demonstrated a statistically significant reduction in the risk of death. Importantly, these benefits appeared independent of PD-L1 status, suggesting that more potent HER2 inhibition may mitigate the dependence on PD-L1 expression observed with standard ICIs.^7^

However, a critical limitation of the HERIZON-GEA-01 trial is the lack of a direct head-to-head comparison with the current standard of care: the KEYNOTE-811 regimen (chemotherapy, trastuzumab, and pembrolizumab). As such, the relative efficacy of zanidatamab-based combinations compared to immunotherapy-augmented trastuzumab remains an open and clinically significant question.

## Methods

We performed a reconstructed individual patient data (IPD) pooled analysis of phase II/III clinical trials evaluating first-line targeted and immunotherapy-based combinations for HER2-positive advanced gastric or GEJ adenocarcinoma. This study followed the Preferred Reporting Items for Systematic Reviews and Meta-Analyses (PRISMA) and Cochrane guidelines for IPD analysis (Supplementary Methods).

Studies were selected based on the following criteria: (1) single-arm or randomized phase II or III clinical trials; (2) patients with HER2-positive metastatic gastric/GEJ adenocarcinoma previously untreated in this setting; (3) treated with experimental or control arms involving (a) Trastuzumab + Chemotherapy (ToGA regimen), (b) Pembrolizumab + Trastuzumab + Chemotherapy (KEYNOTE-811 regimen), or (c) Zanidatamab + Chemotherapy ± Tislelizumab (HERIZON-GEA-01 regimens); eligible chemotherapy regimens were fluoropyrimidine-platinum doublets, including CAPOX, FOLFOX, and Cisplatin + 5-fluoruracil; and (4) availability of Kaplan-Meier (KM) plots for OS and PFS with associated “number-at-risk” tables.

Across all included regimens, the chemotherapy backbone was a fluoropyrimidine plus platinum doublet (capecitabine or 5-fluorouracil combined with oxaliplatin or cisplatin). No crossover between treatment arms was permitted in randomized studies (either KEYNOTE-811 or HERIZON-GEA-01).

A systematic search was conducted across PubMed, Scopus, and major conference libraries (ASCO and ESMO) for trials published or presented between January 1, 2010, and December, 2025. To facilitate a comparative analysis in the absence of head-to-head data, IPD were reconstructed from published KM curves. We utilized the graphical reconstructive algorithm described by Guyot et al. as previously reported.^8^ Digital coordinates were extracted from high-resolution KM plots. These data, combined with the number-at-risk reported in the original manuscripts, were used to recreate a patient-level dataset for each treatment arm. Reconstruction accuracy was verified by comparing the hazard ratios (HR) and median survival times derived from the reconstructed data with those reported in the original publications. Pooled OS and PFS curves were estimated using the Kaplan-Meier method. Cox proportional hazards regression models were used to estimate HRs with 95% CIs, with the specific clinical trial included as a random variable (frailty model). Moreover, to account for potential non-proportional hazards and differences in follow-up duration between trials, restricted mean survival time (RMST) analysis was performed, with time points *t* set at 12, 24, and 36 months. Relative risk of progression/death was expressed as RMST ratios between treatment arms (ratio >1 indicating improved survival).

The primary objective was to compare the efficacy of zanidatamab-based combinations with the current standard of care (trastuzumab + pembrolizumab + chemotherapy) in terms of PFS and OS. For an exploratory PD-L1 CPS-stratified analysis, Kaplan-Meier curves for the CPS ≥1 subgroup were available for both KEYNOTE-811 and HERIZON-GEA-01, whereas curves for the CPS <1 subgroup were reported only for HERIZON-GEA-01. To reconstruct the unreported KEYNOTE-811 CPS <1 subgroup, we applied the KMSubtraction algorithm,^9^ which matches each patient of the reported subgroup to a patient in the parent arm using minimum-cost bipartite (Hungarian) matching on event and censoring times; the unmatched parent-arm patients approximate the complementary subgroup. PFS and OS within each CPS stratum were then estimated by the Kaplan-Meier method, and treatment effects were compared using Cox proportional hazards models.

All analyses were conducted using the R statistical language (version 4.5.0, 2025-04-11) in Positron (Version: 2026.03.0).

## Results

**Figure 1.**
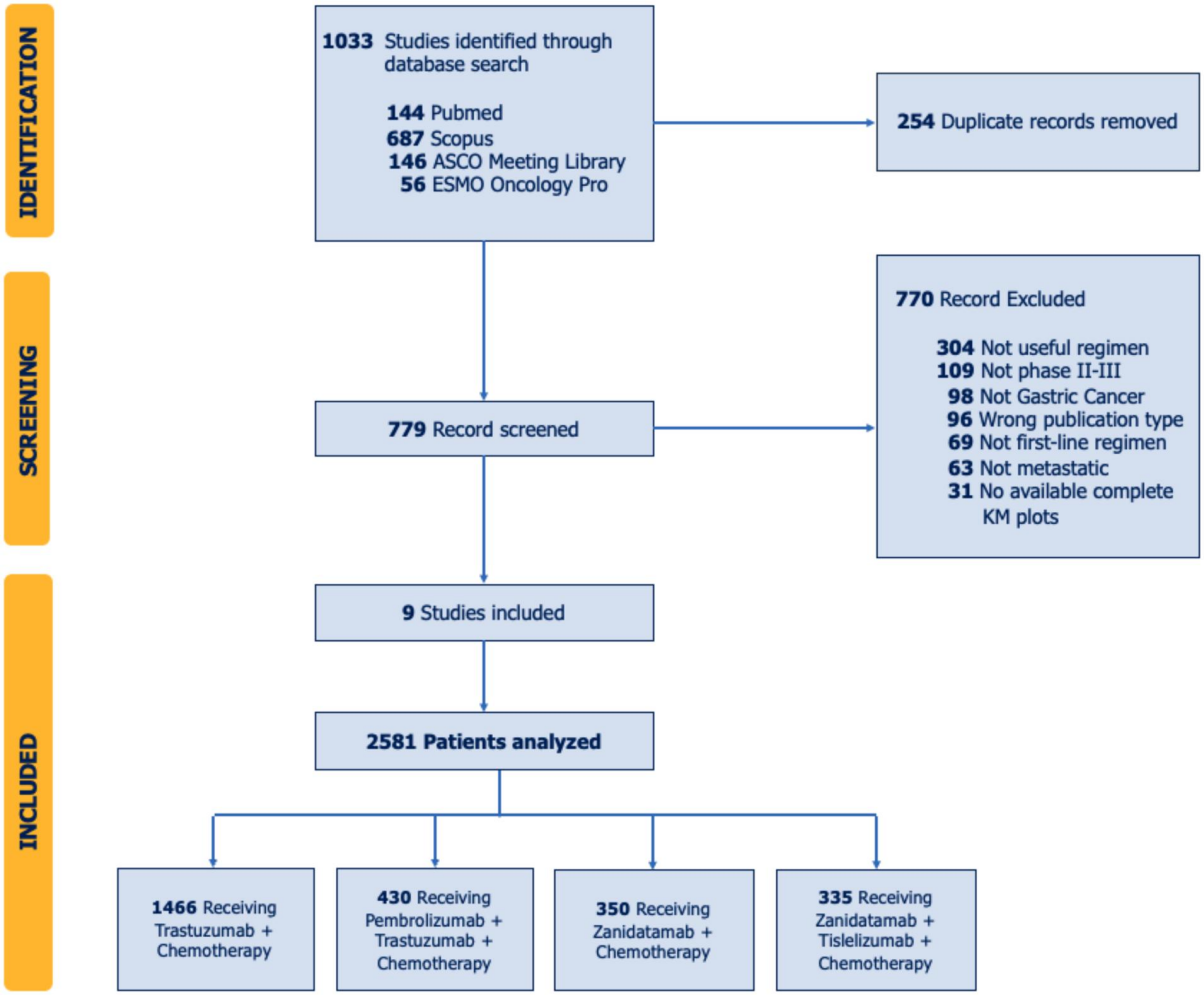
PRISMA flow diagram. Abbreviations: PRISMA = Preferred Reporting Items for Systematic Reviews and Meta-Analyses.

Nine trials were included after screening (Figure 1).^1^^,^^4^^,^^5^^,^^10–16^ After deduplication and screening, 9 trials were finally selected for the analysis. Of these, 4 were phase II trials; for the Jacob and Heloise studies, only the control arm (Trastuzumab + Chemotherapy) was used, while all arms were included from the remaining studies, namely 5 Trastuzumab + Chemotherapy arms (total *n* = 1466), 3 Pembrolizumab + Trastuzumab + Chemotherapy (total *n* = 430), 2 Zanidatamab + Chemotherapy (total *n* = 350) and 2 Zanidatamab + Chemotherapy + Tislelizumab (total *n* = 335) arms. The characteristics of the studies are summarized in Table 1. The risk of bias analysis yielded a low risk for all studies (Supplementary Methods). The graphical reconstructive algorithm yielded patient-level data that derived similar median PFS, OS, and HRs to original trials.

**Table 1.** Characteristics of clinical trials included in the reconstructed pooled analysis.

Name of the trial	ToGA	HELOISE	Janjigian et al^12^	PANTHERA	JACOB	KEYNOTE-811	Elimova et al^10^	HERIZON-GEA-01	Keun-Wook Lee et al^14^
**NCT identifier**	NCT01041404	NCT01450696	NCT02954536	NCT02901301	NCT01774786	NCT03615326	NCT03929666	NCT05152147	NCT04276493
**Year of publication**	2010	2017	2020	2021	2022	2024	2025	2026	2026
**Phase**	III	IIIb	II	Ib/II	III	III	II	III	Ib/II
**Geographic area** **(Asian %)**	Global 51% (Exp)/54% (Ctrl)	Global 51% (Exp)/29.8% (Ctrl)	United States 5%	South Korea 100%	Global 47% (Exp)/47% (Ctrl)	Global 34% (Exp)/35% (Ctrl)	Global 37%	Global 52.6% (Zani + TIS + CT)/53.6% (Zani + CT)/53.6% (Tras + CT)	China; South Korea 100%
**Blinding**	Open-label	Open-label	Open-label	Open-label	Double-blind	Double-blind	Open-label	Open-label	Open-label
**Randomization**	1:1	1:1	Not applicable (single-arm)	Not applicable (single-arm)	1:1	1:1	Not applicable (single-arm)	1:1:1	Not applicable (single-arm)
**Primary endpoint**	OS	OS	PFS	ORR	OS	PFS; OS	ORR	PFS; OS	ORR
**Number of patients**	594	248	37	43	780	698	46	914	33
**Control arm**	Chemotherapy (FP + cisplatin)	Trastuzumab + chemotherapy (FP + cisplatin)	Not applicable (single-arm)	Not applicable (single-arm)	Placebo + trastuzumab + chemotherapy (FP + cisplatin)	Placebo + trastuzumab + chemotherapy (FP + oxaliplatin/cisplatin	Not applicable (single-arm)	Trastuzumab + chemotherapy (FP + oxaliplatin/cisplatin)	Not applicable (single-arm)
**Experimental arm**	Trastuzumab + chemotherapy (FP + cisplatin)	Loading-dose trastuzumab 8 mg/kg followed by HD trastuzumab maintenance 10 mg/kg every 3 weeks + chemotherapy (FP + cisplatin)	Pembrolizumab + trastuzumab + chemotherapy (FP + oxaliplatin/cisplatin)	Pembrolizumab + trastuzumab + chemotherapy (FP + oxaliplatin/cisplatin)	Pertuzumab + trastuzumab + chemotherapy (FP + cisplatin)	Pembrolizumab + trastuzumab + chemotherapy (FP + oxaliplatin/cisplatin)	Zanidatamab + chemotherapy + (FP + oxaliplatin/cisplatin)	Zanidatamab + chemotherapy (FP + oxaliplatin/cisplatin) ± tislelizumab	Zanidatamab + tislelizumab + chemotherapy (FP + oxaliplatin)
**PD-L1 distribution**	Not reported	Not reported	CPS <1: 35% CPS ≥ 1: 38% Not available: 27%	CPS <1: 39.5% CPS ≥ 1: 50.4% Not available: 11.6%	Not reported	CPS <1: 15% CPS ≥ 1: 85% (Exp)/CPS <1: 15% CPS ≥ 1: 85% (Ctrl)	Not reported	TAP score <1: 29.8% TAP score ≥ 1: 61.9 Not available: 8.3% (Zani + TIS + CT)/TAP score <1: 35.5% TAP score ≥ 1: 58.6% Not available: 5.9% (Zani + CT) /TAP score <1: 31.8% TAP score ≥ 1: 61% Not available: 5.9% (Tras + CT)	CPS <1: 24.2% CPS ≥ 1: 72.7%
**Median OS for each arm**	13.8 months (Exp)/11.1 months (Ctrl)	10.6 (Exp)/12.5 (Ctrl)	27.3 months (Exp)	19.3 months (Exp)	18.1 months (Exp)/14.2 months (Ctrl)	20.0 months (Exp)/16.8 months (Ctrl)	36.5 months (Exp)	26.4 months (Zani + TIS + CT)/24.4 months (Zani + CT)/19.2 months (Tras + CT)	32.4 months (Exp)
**Median PFS for each arm**	6.7 months (Exp)/5.5 months (Ctrl)	5.6 (Exp)/5.7 (Ctrl)	13.0 months (Exp)	8.6 months (Exp)	8.5 months (Exp)/7.2 months (Ctrl)	10.0 months (Exp)/8.1 months (Ctrl)	12.5 months (Exp)	12.4 months (Zani + TIS + CT)/12.4 months (Zani + CT)/8.1 months (Tras + CT)	16.7 months (Exp)
**Subsequent anti-HER2 therapy**	2% (Exp)/2% (Ctrl)	11% (Exp)/16% (Ctrl)	23%	Not reported	8.5% (Exp)/9.4% (Ctrl)	16% (Exp)/18% (Ctrl)	41%	16% (Zani + TIS + CT)/23% (Zani + CT)/29.2% (Tras + CT)	Not reported

Abbreviations: 5-FU: 5-fluorouracil; CAPOX: capecitabine + oxaliplatin; CT: chemotherapy; Ctrl: control arm; DLTs: dose-limiting toxicities; Exp: experimental arm; FP: fluoropyrimidine + platinum; HD: high dose; NCT: National Clinical Trial (ClinicalTrials.gov identifier); ORR: objective response rate; OS: overall survival; PFS: progression-free survival; RP2D: recommended phase II dose; SoC: standard of care; TIS: tislelizumab; Tras: trastuzumab; Zani: zanidatamab.

**Figure 2.**
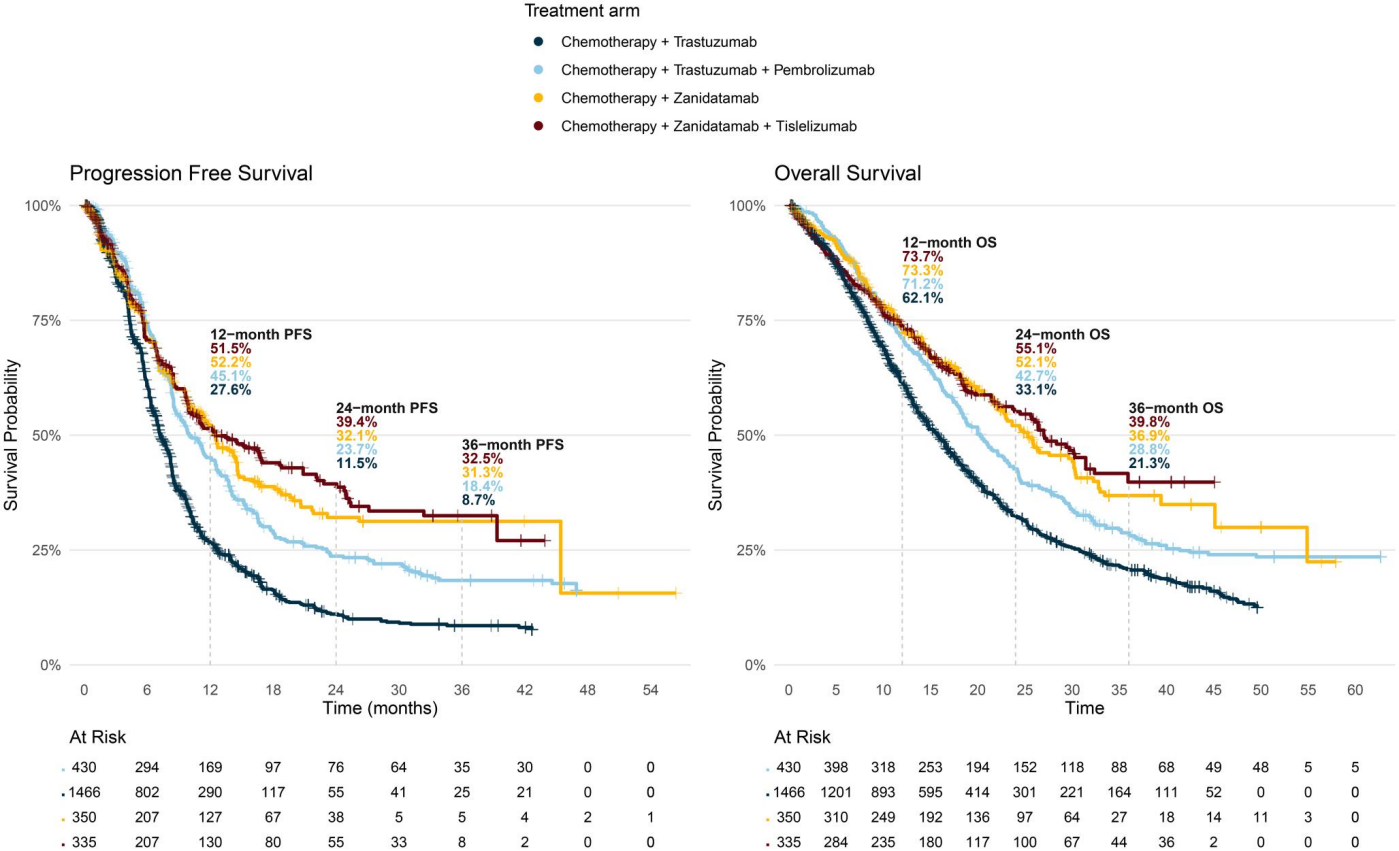
Reconstructed Kaplan-Meier survival curves according to first-line treatment strategy. (A) Progression-free survival and (B) overall survival. Survival outcomes are shown for 4 treatment strategies: chemotherapy plus trastuzumab; chemotherapy plus trastuzumab and pembrolizumab; chemotherapy plus zanidatamab; and chemotherapy plus zanidatamab and tislelizumab. Abbreviations: OS = overall survival; PFS = progression-free survival.

Zanidatamab-containing regimens were associated with the longest progression-free survival. Median PFS was 12.5 months (95% CI, 10.8-14.6) with chemotherapy plus zanidatamab and 12.5 months (95% CI, 9.9-18.6) with chemotherapy plus zanidatamab and tislelizumab, compared with 10.0 months (95% CI, 8.8-12.1) for chemotherapy, trastuzumab and pembrolizumab and 7.3 months (95% CI, 7.0-7.8) for chemotherapy plus trastuzumab alone (Figure 2A, Table S1). In Cox proportional hazards models adjusted for trial of origin and using chemotherapy, trastuzumab and pembrolizumab as the reference regimen, chemotherapy plus trastuzumab alone was associated with significantly worse PFS (HR, 1.41, 95% CI, 1.21-1.64; *P* < .001). Chemotherapy plus zanidatamab showed a numerically lower hazard of progression (HR, 0.87, 95% CI, 0.69-1.10; *P* = .238), while chemotherapy plus zanidatamab and tislelizumab demonstrated a statistically significant improvement (HR, 0.78, 95% CI, 0.62-0.99; *P* = .042).

RMST analyses were consistent with these findings. Compared with the pembrolizumab-containing regimen, trastuzumab-based chemotherapy alone showed significantly shorter PFS across time horizons, including at 12 months (RMST ratio 1.18, *P* < .001), 24 months (ratio 1.32, *P* < .001), and 36 months (ratio 1.42, *P* < .001). In contrast, zanidatamab-containing regimens showed comparable or improved long-term outcomes. While differences versus the pembrolizumab regimen were not significant at earlier time points, chemotherapy plus zanidatamab and tislelizumab showed significantly longer PFS at 24 months (RMST ratio 0.88, *P* = .010) and 36 months (ratio 0.82, *P* < .001), indicating a sustained benefit over time (Table S2).

Median OS also differed across treatment strategies. The longest median OS was observed with chemotherapy plus zanidatamab and tislelizumab (27.0 months, 95% CI, 23.9-32.4), followed by chemotherapy plus zanidatamab (25.3 months, 95% CI, 22.7-30.2). Median OS was 20.3 months (95% CI, 18.4-22.9) with chemotherapy, trastuzumab and pembrolizumab and 15.8 months (95% CI, 14.7-16.7) with chemotherapy plus trastuzumab alone (Figure 2B, Table S1).

In Cox models, chemotherapy plus trastuzumab alone was associated with inferior OS compared with the pembrolizumab-containing regimen (HR, 1.24, 95% CI, 1.06-1.45; *P* = .008). Chemotherapy plus zanidatamab (HR, 0.91, 95% CI, 0.70-1.18; *P* = .473) and chemotherapy plus zanidatamab and tislelizumab (HR, 0.84, 95% CI, 0.65-1.09; *P* = .180) showed numerically lower hazards for death but did not reach statistical significance.

RMST analyses supported these findings. Compared with trastuzumab-based chemotherapy, the pembrolizumab regimen demonstrated significantly longer OS at 12 months (RMST ratio 1.07, *P* < .001), 24 months (ratio 1.13, *P* < .001), and 36 months (ratio 1.17, *P* < .001). Zanidatamab-containing regimens showed comparable survival to the pembrolizumab regimen at earlier time points, but significantly longer long-term survival. At 36 months, both chemotherapy plus zanidatamab (RMST ratio 0.92, *P* = .04) and chemotherapy plus zanidatamab and tislelizumab (ratio 0.91, *P* = .03) demonstrated longer survival compared with the pembrolizumab regimen. Differences between the 2 zanidatamab-based strategies were not significant (Table S3).

In an exploratory PD-L1 CPS-stratified analysis (Figure S1 [see online supplementary material for a color version of this figure], Tables S4-S6), chemotherapy plus zanidatamab and tislelizumab was associated with longer PFS and OS than chemotherapy plus trastuzumab and pembrolizumab in both CPS ≥ 1 and CPS < 1 subgroups. Using pembrolizumab in the CPS ≥ 1 stratum as the common Cox reference, zanidatamab plus tislelizumab significantly reduced the hazard of progression in CPS ≥ 1 (HR, 0.77; *P* = .040) and to a similar or greater extent in CPS < 1 (HR, 0.70; *P* = .037), whereas pembrolizumab in CPS < 1 did not differ from the reference (PFS HR, 1.10, *P* = .597; OS HR, 1.31, *P* = .112). For overall survival, the effect of zanidatamab plus tislelizumab was more pronounced in the CPS < 1 stratum (HR, 0.54; *P* = .002) than in CPS ≥ 1 (HR, 0.80; *P* = .094). Restricted mean survival time contrasts favored zanidatamab plus tislelizumab at 36 months in both strata for PFS and, for OS, in the CPS < 1 stratum only.

## Discussion

In this reconstructed patient-level pooled analysis, zanidatamab-based regimens compared favorably with trastuzumab-based standards in first-line HER2-positive advanced gastric/GEJ adenocarcinoma. The most important signal was not simply a numerical improvement in medians, but the shape and persistence of benefit over time. Relative to chemotherapy, trastuzumab, and pembrolizumab, zanidatamab-containing combinations produced the most consistent gains in PFS, with the triplet of zanidatamab, tislelizumab, and chemotherapy achieving a significant reduction in the hazard of progression. For OS, RMST analyses suggested that any benefit from zanidatamab-based therapy may emerge more clearly in the longer term, particularly at 36 months. Taken together, these findings support the idea that more potent HER2 blockade may alter the natural history of the disease beyond what is captured by median survival alone.

This point is clinically relevant because the current first-line benchmark is no longer trastuzumab plus chemotherapy, but trastuzumab, chemotherapy, and pembrolizumab for tumors with a PD-L1 CPS ≥1. The key unanswered question is therefore not whether zanidatamab is superior to historical trastuzumab-based therapy, but whether intensified HER2 targeting can compete with or even outperform the immunotherapy-augmented standard. In other words, these data suggest that in HER2-positive gastric cancer, the quality of HER2 inhibition itself may remain a major determinant of outcome, even in the era of checkpoint inhibition.

At the same time, the results also suggest a more nuanced interpretation of the role of immunotherapy. The OS advantage of the addition of tislelizumab to zanidatamab and chemotherapy remained less definitive and the difference versus zanidatamab plus chemotherapy alone was modest. This raises the possibility that once HER2 inhibition is substantially intensified, the incremental contribution of PD-1 blockade may become smaller, delayed, or restricted to selected biological subsets. That hypothesis is provocative because it differs from the KEYNOTE-811 paradigm, where the benefit of pembrolizumab appears largely concentrated in PD-L1 CPS ≥1 tumors. By contrast, HERIZON-GEA-01^11^ suggested activity irrespective of PD-L1 status, implying that stronger HER2 targeting could partly overcome the dependence on immune enrichment that characterizes trastuzumab-based immunotherapy combinations. Whether zanidatamab can reduce the predictive importance of PD-L1 is therefore one of the most important mechanistic and clinical questions raised by these data.

Consistent with, but not confirming, this hypothesis, our exploratory CPS-stratified analysis showed a comparable direction and magnitude of benefit from chemotherapy plus zanidatamab and tislelizumab over the pembrolizumab-containing regimen in both the CPS ≥1 and CPS <1 subgroups. However, these observations should be regarded as hypothesis-generating and require confirmation in biomarker-stratified randomized comparisons before informing treatment selection.

The main strategic challenge is that first-line optimization can no longer be considered independently from subsequent lines of therapy. Trastuzumab deruxtecan (T-DXd) has already changed the post-progression landscape, currently being the standard second-line regimen for trastuzumab-pretreated cases.^17^ Although detailed data are lacking, it is likely that most patients enrolled in the trials included in our analysis did not receive T-DXd after first-line progression, meaning that the survival outcomes observed may not fully reflect contemporary treatment sequences. In this context, the value of a zanidatamab-based first-line strategy will depend not only on its ability to improve initial PFS, but also on whether it preserves or compromises sensitivity to subsequent antibody-drug conjugates. A regimen that maximizes early tumor control may still be suboptimal if it promotes resistant clones, alters HER2 expression, or reduces the activity of T-DXd in later lines. Conversely, deeper early HER2 suppression may delay symptomatic progression and increase the proportion of patients able to receive second-line ADC therapy. Thus, the clinically relevant endpoint may extend beyond first progression to the performance of the entire treatment sequence.

This issue becomes even more complex as T-DXd is being evaluated in the first-line setting in trials such as DESTINY-Gastric-03 (NCT04379596), DESTINY-Gastric-05 (NCT06731478), and ARTEMIDE-Gastric-01 (NCT06764875). If T-DXd moves earlier in the treatment paradigm, the field will need to determine whether the priority should be maximal upfront cytoreduction with the most active anti-HER2 agent or preservation of complementary HER2-targeting mechanisms across sequential lines. Zanidatamab, trastuzumab-based chemoimmunotherapy, and T-DXd engage HER2 through distinct mechanisms, and their optimal positioning will likely depend on tumor biology, HER2 heterogeneity, PD-L1 status, and mechanisms of resistance emerging during treatment.^12^

Beyond HER2 and PD-L1, additional biomarkers are likely to modulate response to intensified HER2 blockade. A recent study by Yoshino et al. demonstrated that pretreatment intratumoral HER2 heterogeneity fundamentally limits the efficacy of later-line antibody-drug conjugates such as T-DXd: using an optimal cutoff of 79% HER2-positive cells, patients in the HER2-homogeneous group achieved significantly longer PFS (6.3 vs 3.1 months) and OS (9.8 vs 6.0 months) than those with heterogeneous expression.^18^ This divergence is strongly linked to clonal selection under therapeutic pressure, with loss of HER2 expression documented in 25%-69% of patients following first-line trastuzumab-based therapy, predominantly affecting initially heterogeneous or low-expressing clones. Serial ctDNA tracking of ERBB2 copy-number fluctuations has emerged as a promising tool to monitor real-time resistance and clonal evolution non-invasively. Intensified HER2 blockade strategies, such as the biparatopic antibody zanidatamab, may exploit distinct mechanisms to overcome these biological limitations. Mechanistic data from Weisser et al.^19^ show that zanidatamab binds non-overlapping extracellular domains (ECD2 and ECD4) in trans, bridging adjacent HER2 molecules, driving macroscale spatial reorganization into polarized cell-surface “HER2 caps” and large nanoscale clusters, and inducing robust receptor internalization, total HER2 degradation, and down-regulation of downstream pHER3 and pAKT signaling. Whereas conventional anti-HER2 monospecific antibodies fail to engage the complement cascade in human serum, the unique geometry of zanidatamab-induced clusters triggers potent complement-dependent cytotoxicity in HER2-high cells, and its efficacy is further modulated by immune microenvironment features that orchestrate antibody-dependent cellular cytotoxicity and phagocytosis. Whether these determinants—HER2 heterogeneity, ctDNA-tracked clonal evolution, complement engagement, and immune-microenvironment features—reshape response to zanidatamab in first-line HER2-positive gastric cancer remains an open question that will need to be addressed in dedicated translational studies.

Our study has the intrinsic limitations of an indirect reconstructed IPD analysis, including the residual imprecision inherent to patient-level reconstruction from published Kaplan-Meier curves and the lack of individual patient covariates. Although 9 trials were included in the reconstructed pooled analysis to provide historical and quantitative context, the central indirect comparison that motivates the clinical question of this work is between the 2 contemporary regimens: chemotherapy + trastuzumab + pembrolizumab (KEYNOTE-811, the current first-line standard for CPS ≥1 tumors) and zanidatamab-based combinations (HERIZON-GEA-01). Because this KEYNOTE-811 versus HERIZON-GEA-01 comparison drives the interpretation of the analysis, several between-trial differences warrant explicit consideration. First, the 2 trials differed in the distribution of PD-L1 expression: approximately 85% of patients in KEYNOTE-811 had a CPS ≥1, compared with approximately 72% in HERIZON-GEA-01. Second, geographic recruitment was imbalanced, with a higher proportion of Asian patients in HERIZON-GEA-01 (53%) than in KEYNOTE-811 (34%); this population has previously shown different survival trajectories in gastric cancer trials, and the imbalance may therefore contribute to between-trial heterogeneity. Third, post-progression access to subsequent anti-HER2 therapy differed markedly: in HERIZON-GEA-01, 12.9% of patients on the zanidatamab + tislelizumab arm and 23% of those on the zanidatamab arm received any anti-HER2 therapy after progression (with a HER2-directed antibody-drug conjugate in 5.6% and 11.2%, respectively, and trastuzumab in 8.3% and 14.5%), whereas the corresponding proportion was 16% in KEYNOTE-811. In addition, because OS data for the zanidatamab cohorts are still maturing, the long-term clinical applicability of these findings will become more definitive with extended follow-up. These caveats prevent definitive treatment ranking. Nevertheless, in the absence of direct randomized comparisons, this approach provides a clinically useful signal: zanidatamab-based combinations appear at least competitive with the KEYNOTE-811 standard and may confer a more durable disease-control advantage.

In conclusion, our analysis suggests that zanidatamab-based first-line treatment may represent a meaningful advance in HER2-positive gastric cancer, particularly by extending PFS and possibly improving long-term survival. The next challenge for the field is no longer simply to intensify first-line therapy, but to determine how best to integrate bispecific antibodies, checkpoint inhibitors, and ADCs across the full treatment continuum. As T-DXd moves into earlier lines, the central question will be whether the optimal strategy is to maximize HER2 inhibition immediately, or to design a sequence that preserves the effectiveness of multiple HER2-directed agents over time.

## Supplementary Material

oyag270_Supplementary_Data

## Data Availability

Efficacy data are available in the original publications of each included clinical trial. Code for data reconstruction and analysis is available upon reasonable request to the corresponding author.
